# The Vaccine World of COVID-19: India’s Contribution

**DOI:** 10.3390/vaccines10111943

**Published:** 2022-11-17

**Authors:** Vivek P. Chavda, Disha R. Vihol, Hetvi K. Solanki, Vasso Apostolopoulos

**Affiliations:** 1 Department of Pharmaceutics and Pharmaceutical Technology, L M College of Pharmacy, Ahmedabad 380008, Gujarat, India; 2 Pharmacy Section, Griffith University, Gold Coast, QLD 4215, Australia; 3 Institute for Health and Sport, Victoria University, Melbourne, VIC 3021, Australia; 4 Immunology Program, Australian Institute for Musculoskeletal Science (AIMSS), Melbourne, VIC 3021, Australia

**Keywords:** SARS-CoV-2, COVID-19, Covaxin, Covishield, vaccine, clinical trials

## Abstract

The severe acute respiratory syndrome coronavirus 2 (SARS-CoV-2) eruption has left not only illness and mortality in its wake, but also an overwhelming threat to health policy, human regality, food security, and struggle worldwide. The accessibility and potential distribution of a protective and successful vaccination to communities throughout the world are being considered now not just, as a potential of overcoming these hurdles, but also as an example of human perseverance in the face of catastrophe. A vaccine is the only tool that can efficaciously deal with the COVID-19 catastrophe. Currently, more than 47 vaccines are permitted for emergency use in distinct parts of the world. India will play a significant role in the development of the high-priced Moderna shots and Pfizer Inc, therefore assisting in the immunization of a large portion of the world. Moreover, many of the internationally researched and developed vaccine laboratories seek manufacturing in Indian firms and companies for efficient and low-cost production of vaccines intending to provide to the world, hence, making India, a major role player during these pandemic times. This review highlights the Indian contribution to the globe for COVID-19 management.

## 1. Introduction

With massive financial expenditures, the COVID-19 pandemic brought about a global joint effort between regulatory authorities, industries, and medical journals with free access to all COVID-related publications. Nowadays, other than variants of concern (VOCs), variants of interests (VOIs), and high consequences, there is one more type of virus, which is known as hybrid variants, recombination between two variants or sub-variants surfaced. To date, three hybrid variants of Omicron are identified XD, XE, and XF [1].

Just like researchers around the globe are striving to develop the finest vaccine to prevent or reduce the severity of the disease, likewise, Indian researchers in partnership with industries have also come forward with some novel vaccination methods against SARS-CoV-2 [2]. More than 60% of all vaccines supplied globally are manufactured in India, yet the $40 billion pharmaceutical industry is not even engaged [3]. Free COVID-19 immunization began in India on 16 January 2021, and the government asked all its citizens to take part in what is thought to be the world’s largest immunization program [4].

**Figure 1:**
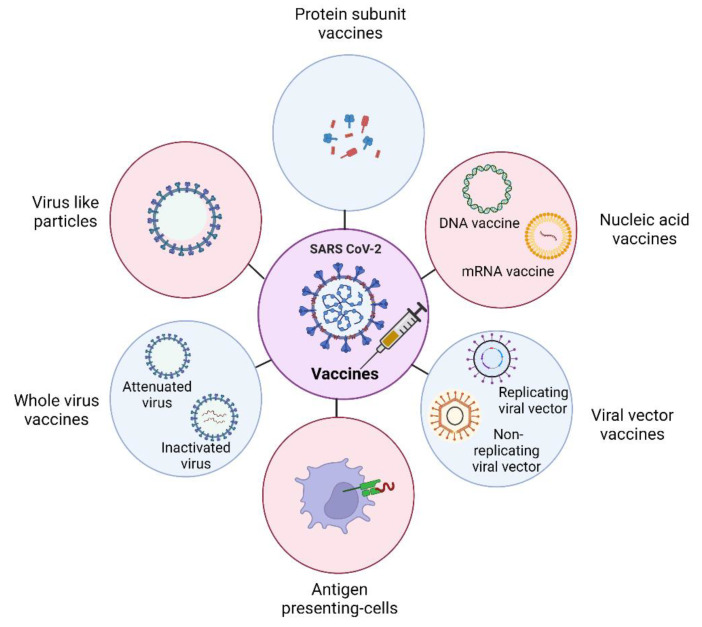
COVID-19 vaccines and their different platforms. (Created with Biorender.com).

COVID-19 has numerous vaccine preferences in development than any other infectious illness has ever had. Various weakened viruses are used in live attenuated vaccines, which are still capable of multiplication and reproduction but do not cause COVID-19 [5]. Other than SARS-CoV-2, there are other life-threatening diseases like “Mucormycosis”, affecting the aged and immunosuppressed people mostly—A cause for caution in COVID-19 [6]. The most exploited component from SARS-CoV-2 for inducing immunogenicity is the spike protein and there are various methods for delivering it to the host cells (Figure 1). One is through inactivated vaccines which do not penetrate and multiply in cells via damaged genetic material of the virus by temperature, chemical, or radiation but are still capable of eliciting an immune response [7]. Another vaccination method is using mRNA technology, which upon entering the host cell guides it to produce harmless virus-specific proteins. In addition, protein subunit vaccines, comprise safe viral proteins, which are internalized by host cells processed, and presented on their surface to stimulate immune responses. Vector-based immunizations, on the other hand, use a modified variant of a virus that is not the same as the one that causes COVID-19, and it is used to deliver the vaccine to host cells [8].

At the time of writing, 220 vaccine candidates and 766 vaccination trials were being conducted in over 78 countries. There are 47 licensed vaccines, 85 in phase III clinical trials, 70 in phase II studies, 55 vaccines in phase I trials, and 9 that are no longer being studied [9]. Globally, more than 12 billion doses of SARS-CoV-2 vaccines have been administered as of 30 September 2022, with more than 65.6% of the world population being fully immunized. Despite differences in immunization efforts among countries, every effort is being taken to prevent the SARS-CoV-2 virus in humans [10,11,12].

## 2. International Collaboration, Development, and Commercialization

Global authorities have acknowledged the need for collaboration and open science in the medical and technology fight against COVID-19 [13], as they have in other major global public health concerns such as climate change and antibiotic resistance [14,15,16]. The call to collaborate has been backed up by key stakeholders [17] and exciting initiatives, such as unprecedented global data sharing [18]—including the genetic sequence of the SARS-CoV-2 [19]; unconventional collaborations [20]; and initiatives for communicating expertise, intellectual property, and innovations [21]. The race to create effective and safe COVID-19 vaccines is one of the crucial areas for collaboration, but also worldwide rivalry (Table 1). Collaborative efforts for vaccine development may include partnerships between commercial industries, hybrids, or public entities, such as public-private partnerships (PPPs). PPPs often comprise both private for-profit and not-for-profit businesses, share resources, and benefits, and are dedicated to enhancing social value and health [22]. They were very useful during the COVID-19 incident [23,24,25,26,27]. Previously, PPPs were utilized to accelerate vaccine development and manufacture or serve developing nations in vital regions [28,29,30,31,32,33].

### 2.1. Covishield^TM^

Serum Institute of India Pvt. Ltd. manufactures ChAdOx1nCoV-19 (recombinant vector) replication-deficient adenoviral vector vaccine (Covishield^TM^), which was developed by Oxford University-AstraZeneca. The Indian Council for Medical Research (ICMR) and the Drugs Controller General of India (DCGI) granted Covishield^TM^ an “at-risk manufacturing and stockpiling license” [40,41]. The vaccine compromise of 5 × 10^10^ virus particles replicated as a deficient chimpanzee adenovirus vector encoding the SARS-CoV-2 Spike glycoprotein produced in the modified human embryonic kidney (HEK)293 cells with excipients such as L-histidine, ethanol, Ethylenediaminetetraacetic acid (EDTA) and its hydrochloride monohydrate form, magnesium chloride hexahydrate, water for injection, polysorbate 80, sucrose, and sodium chloride. The vaccination is injected in two doses of 0.5 mL into the deltoid muscle with a second dose followed at 4–12 weeks [42]. While injecting contraindications may occur in cases like the hypersensitive individual, thrombosis, thrombocytopenia condition, immunocompromised patients, and pregnant and breastfeeding women [43]. Side effects include pain at the site of injection, muscle ache, fatigue, dizziness, headache, fever, irregular heart rhythm, wheezing, swelling of lips, face, or throat, diarrhea, and itchy skin [44].

On 23 April 2020, the University of Oxford, initiated a phase I/II study of the Covishield^TM^ vaccine in 1090 UK participants aged between 18–55 years to assess its efficacy against COVID-19 with an estimated date of completion by 2023 (NCT04324606). In addition, its efficacy and safety were evaluated in a phase I/II randomized placebo-controlled trial in 2130 patients with or without HIV in South Africa (NCT04444674). Then phase II/III was initiated by the University of Oxford on 28 May 2020, which is still in progress in 12,390 healthy UK participants to determine the immunogenicity, safety, and efficacy of Covishield (NCT04400838). Moreover, AstraZeneca and Iqvia Pty Ltd. initiated a phase III double-blind, placebo-controlled study (28 August 2020) in 32,459 participants to determine the immunogenicity, safety, and efficacy of Covishield^TM^ (AZD1222); the study is estimated to complete in early 2023 (NCT04516746).

Covishield^TM^ is mainly effective against B.1.1.7 (Alpha) and B.1.617.2 (Delta), but it shows a higher affinity towards the Delta variant of SARS-CoV-2. In the second wave, Covishield had 63% effectiveness against the B.1.617.2 (Delta) variant [45]. The effectiveness of the vaccination after one dose was significantly lower in people with the delta variation (30.7 percent; 95 percent confidence interval [CI], 25.2 to 35.7) than in people with the alpha variant (48.7 percent; 95 percent CI, 45.5 to 51.7). The efficacy of two doses of the Covishield vaccination was 74.5 percent (95 percent CI, 68.4 to 79.4) in people with the alpha variation and 67.0 percent (95 percent CI, 61.3 to 71.8) in people with the delta variant [46]. Innate immunity, which is equipped with pattern recognition receptors such as Toll-like receptors (TLR), is essential for immune cell activation and the link to antimicrobial adaptive immunity. However, data on the influence of age on innate immunity in response to SARS-CoV2 adenovirus vector vaccines, as well as its relationship with particular immune responses, is limited. The innate immune responses following the initial vaccination are linked with the development of neutralizing antibodies. In comparison to young people, older persons may have faulty innate immune responses to TLR stimulation and a weak or delayed innate immune activation profile following vaccination [47]. In an ongoing phase 1/2, randomized, double-blind, parallel-group, placebo-controlled experiment, the immunogenicity and safety of the AZD1222 (ChAdOx1 nCoV-19) vaccine were assessed in Japanese people (NCT04568031). AZD1222 induced a significant humoral immune response against SARS-CoV-2 and was well tolerated in Japanese subjects, including the elderly [48]. Swanson et al. looked at T-cell responses to the AZD1222/ChAdOx1 nCoV-19 vaccination. Individuals who received two doses of the vaccine displayed polyfunctional CD4+ and CD8+ T cell responses specific to the vaccine-encoded spike protein, according to the researchers. Furthermore, the majority of CD4+ T cell responses were biassed toward TH1 cells. These findings indicate that the AZD1222/ChAdOx1 nCoV-19 vaccination stimulates antiviral T-cell responses in addition to neutralising antibodies [49]. Scleritis as an eye symptom following ChAdOx1nCoV-19 immunization in otherwise healthy people may be known to ophthalmologists [50]. Similarly, Tapadia and colleagues have also reported post-ChAdOx1 nCoV-19 vaccination frontal lobe syndrome [51]. Superficial Vein Thrombosis may be added to the list of side effects linked with the ChAdOx1 nCoV-19, Covishield, and vaccination [52]. Individuals with vaccine-induced immunological thrombotic thrombocytopenia are more likely to have numerous thrombosis sites, most often cerebral venous sinus thrombosis in conjunction with pulmonary embolism and portomesenteric venous thrombosis. Occult thrombosis can be detected using whole-body imaging using contrast-enhanced CT imaging [53]. According to Georg M. N. Behrens and colleagues [54], “Heterologous prime/boost vaccination with a vector-based technique (ChAdOx-1nCov-19, ChAd) followed by an mRNA vaccine (e.g., BNT162b2, BNT) has been shown to be more effective than repeated administration of the same vaccine in eliciting protective immunity. However, there is a dearth of evidence comparing immunity reduction after homologous and heterologous vaccination, as well as the effects of a third vaccine administration following heterologous ChAd/BNT immunization. We provide longitudinal data on ChAd/ChAd (*n* = 41) and ChAd/BNT (*n* = 88) vaccinated people, as well as the impact of a third BNT vaccine. The third immunization significantly boosts fading anti-spike IgG but only resulted in a minor rise in spike-specific CD4 + and CD8 + T cell populations in both groups, compared to cell frequencies already present in the ChAd/BNT group following the second vaccination. More crucially, the third vaccination effectively restores neutralizing antibody responses against the virus’s Alpha, Beta, Gamma, and Delta versions, but not against the B.1.1.529 (Omicron) variant. In conclusion, poor SARS-CoV-2 specific immune responses after homologous ChAd/ChAd vaccination can be compensated for by heterologous BNT vaccination, which may impact vaccine type selection for further immunization boosts”.

### 2.2. Sputnik V

Sputnik V, also known as Gam-COVID-Vac, is the world’s first COVID-19 vaccine to be approved for use in 71 countries, which is manufactured by Moscow’s Gamaleya National Center of Epidemiology and Microbiology. Sputnik V is titled after the first Soviet space satellite, “Sputnik-1”. The vaccine comprises a human adenovirus-based viral vector that employs a heterogeneous boosting method based on two distinct vectors for two vaccine doses i.e., Ad5 and Ad2618 adenoviruses injected into the deltoid muscle for greater immunity sustainability [55,56].

Recombinant adenovirus serotype 26 particles containing the SARS-CoV-2 protein S gene 1.0 ± 0.5 × 10^11^ is the first composition and recombinant adenovirus serotype 5 particles containing the SARS-CoV-2 protein S gene 1.0 ± 0.5 × 10^11^ particles is the second composition of the intramuscular vaccine per dose (0.5 mL) with common excipients like EDTA disodium salt dihydrate, ethanol 95%, sodium chloride, magnesium chloride hexahydrate, sucrose, water for injection, polysorbate 80, and Tris (hydroxymethyl) aminomethane. Does one consists of the component 1 ingredients and 21 days later component 2 vaccine is administered. Contradictions are observed in pregnant and lactating mothers, hypersensitive persons, and persons below 18 years of age. The vaccine is generally considered free from side effects, but a few undesirable effects can be observed in certain cases including chills, fever, myalgia, general discomfort, headache, pain at the site of injection, swelling, nausea, loss of appetite, edema, pruritus, oropharyngeal pain, nasal congestion, and sore throat. In a non-randomized study of 38 healthy participants, safety and tolerability were assessed using prime-boost immunization (NCT04436471). Then a phase III study (7 September 2020), with 33,758 participants randomized in parallel assignments for evaluating the vaccine’s immunogenicity, safety, and efficacy against the SARS-CoV-2 infection (NCT04530396) was completed in May 2021. Furthermore, 1600 subjects were tested to determine the immunogenicity, and Dr. Reddy’s Laboratories Limited in India, carried out phase II/III safety of the Gam-COVID-Vac combined vector vaccine against SARS-CoV-2 infection (NCT04640233; 30 November 2020–September 2021).

From 5 January–1 March 2021, for the identification of vaccine safety, Hospital Italiano de Buenos Aires, Argentina (NCT04738435) carried out an observational study on 600 participants in health personnel of private effectors. Another observational study was carried out on 82 participants for assessing the safety, immunogenicity, and reactogenicity of the vaccine against COVID-19 (NCT04871841) residing in Kazakhstan on 5 April 2021 by Karaganda Medical University and McMaster University. The outcome of this study is that following the first dose, adverse effects like swelling, itching, and redness at the site of injection and systemic adverse effects such as headache, joint pain, nausea, vomiting, fever, and chills were observed. Change in the titers of mucosal SARS-CoV-2 binding antibodies, systemic and mucosal anti-SARS-CoV-2 neutralizing antibodies, and systemic cytokine stimulation were noted [57].

Data analyses show that the vaccine is 97.6% effective in generating protective neutralizing antibodies against Alpha (B.1.1.7), Beta (B.1.351), Gamma (P.1), Delta (B.1.617.2, and B.1.617.3), and variants B.1.1.317 and B.1.1.141 with mutations in the receptor-binding domain [46].

## 3. Covaxin, by Bharat Biotech, India

Bharat Biotech International Limited developed the Bharat Biotech COVID-19 vaccine (COVAXIN^®^), in collaboration with the National Institute of Virology; this is India’s first indigenous COVID-19 vaccine [58]. Covaxin is a Vero cell-derived inactivated viral vaccine incorporating 6 µg of whole attenuated SARS-CoV-2 viral antigen (Strain: NIV-2020-770) with excipients like 2-phenoxyethanol, aluminum hydroxide gel, imidazoquinolinone, and phosphate buffer saline for marking up. As an adjuvant, the immobilized virus is combined with Alhydroxiquim-II (Algel-IMDG), which is chemisorbed imidazoquinoline onto aluminum hydroxide gel, to increase immune response and provide longer-lasting immunity. The intramuscular delivery of the vaccine is given in a series of 2 doses 4 weeks apart from the upper arm into the deltoid muscle for restricted use in an emergency in individuals aged 18 years and older of age [59,60].

Vaccine-related side effects like body aches, pain, headache, fever, swelling, nausea, vomiting, rashes, itching, and redness at the site of injection have been reported during clinical trials. The vaccine is not contraindicated in the case of individuals taking anticoagulation therapy consisting of aspirin, clopidogrel, warfarin, or a new anticoagulant rivaroxaban or apixaban, but in a few cases, stoppage of bleeding may take a while following vaccination and can lead to bruising on the upper arm. In such cases, further precautions are required, and a fine needle of 23–25 gauge is utilized, followed by 2 min of hard pressure on the spot. In the case of patients taking immunosuppressive medications, there may be lowered effectiveness of the vaccine [59].

A phase I clinical trial was carried out on 300 volunteers for assessing the safety and immunogenicity at 11 Indian hospitals. Then, in the same manner, a phase II clinical trial with 2-doses of Covaxin was carried out in 380 volunteers at 9 hospitals across India (NCT04471519). On 16 November 2020, the Indian Council of Medical Research (ICMR) and Bharat Biotech Ltd. launched phase III studies of Covaxin with 25,800 participants across 25 facilities in India with 78% vaccine efficacy results (CTRI/2020/11/028976; NCT04641481) [61,62]. On 29 December 2021, it was suggested that Covaxin be used for ages 2–18 years, which was backed up by a phase II/III study across six hospitals in India assessed for the age of de-escalation study with safety, reactogenicity, and immunogenicity parameters, and the results demonstrated an enhanced efficacy compared to the adults, suggesting the use of the vaccine for children and teenagers (CTRI/2021/05/033752). Despite the strong immune responses noted, the vaccine comes with a drawback of declining efficacy (like all other COVID-19 vaccines) after 6 months of vaccination and raises a need for a booster dose to maintain immunity. After receiving re-consent from 184 previously vaccinated subjects to receive a third dose of vaccination or placebo on day 215, a third dose was introduced into the current phase II study (NCT04471519). The findings suggested a 19- to 97-fold increase in antibodies against COVID-19 after a third dose, proving that the booster dose is effective, safe, and necessary to ensure persistent immunity [63].

**Figure 2:**
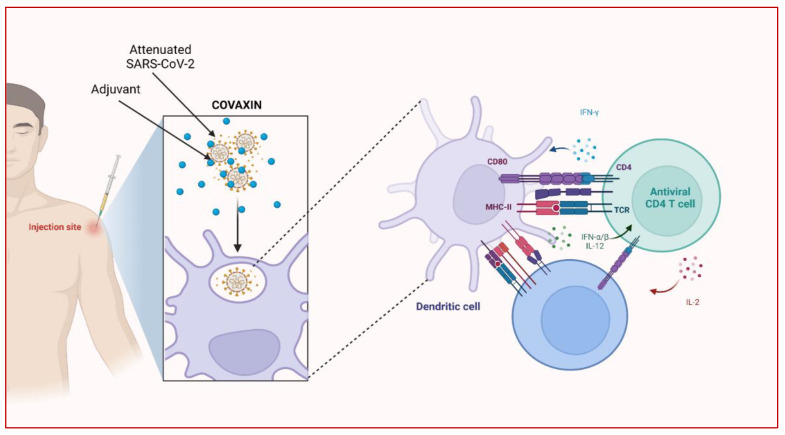
Working principle of COVAXIN against the SARS CoV-2 and its immune response. (Created with Biorender.com).

Covaxin increases anti-SARS-CoV-2 IgA/IgG antibodies against both nucleocapsid (N) and spike (S) antigens at M1, M2, M3, M4, M6, and M12 compared to M0 [64]. It increases the levels of type-17, type-1, and other pro-inflammatory cytokines as well as raising plasma levels of IL-3, and IL-7 while decreasing IL-25, IL-33, GM-CSF, IL-1Ra, IFN levels [65]. It also stimulates an increased humoral immune response that lasts at least 12 months after vaccination against most viral variants (Figure 2) [64]. In addition, it is 78% effective against the severity of COVID-19 in 14 days or more after the second dosage. In adults under the age of 60, efficacy was demonstrated at about 79%, and for those aged over 60, it was 68%. Moreover, its efficacy against asymptomatic infection was 64% [66]. The vaccine has proven its effectiveness against various variants such as B.1.1.7 (Alpha), B.1.351 (Beta), P.1-B.1.1.28 (Gamma), B.1.617.2 (Delta), P.2-B.1.1.28 (Zeta), B.1.617 (Kappa), with the efficacy of 65.2% [67]. Further, Covaxin has shown about a 90% neutralizing effect on the recent variant B.1.1.529 (Omicron) [68,69,70]. Currently, the vaccine is approved in India and other 14 countries [71].

## 4. Vaccines under Different Stages of Clinical Development in India

### 4.1. ZyCoV-D

ZyCoV-D (Novel CoronaVirus-2019-nCov vaccine), by Cadila Healthcare, Ahmedabad, is relying on the novel plasmid DNA vaccine technology, where a recombinant plasmid is employed as a vector to deliver DNA into cells. This technology is yet to be approved for usage in the general population [72]. A phase I study for this vaccine formulation in 150 participants aged 18–55 (CTRI/2021/03/032051), showed safety and tolerability, and a phase II study in over 1048 participants across 9 sites (CTRI/2020/07/026352). In addition, a phase III trial is being conducted to evaluate its efficacy, safety, and immunogenicity against SARS-CoV-2 in 28,216 participants selected in a randomized manner (CTRI/2021/01/030416). The route of injection is via the intradermal route using a needle-free injector and is scheduled in 3 doses of 2 mL each to be given at an interval of 28 days (day 0, day 28, and day 56) [73]. The vaccine consists of a DNA plasmid constructed with the spike gene with a phosphate buffer and can be injected into individuals ≥ 12 years old. The vaccine is generally categorized as safe for use, with some side effects such as headache, muscle pain, fever, nausea, and diarrhea [74].

### 4.2. NVX-CoV2373

A protein-based COVID-19 vaccine candidate has been developed by Novavax, a US-based company, using a new nanoparticle technology using the gene sequence from the SARS-CoV-2 beta coronavirus strain [75]. The vaccine utilizes nanosized Matrix-M, as an adjuvant to enhance antigen representation and boost immune responses by stimulating high levels of neutralizing antibodies. Here, a modified coronavirus spike protein gene termed baculovirus is engineered and cultures are prepared in Sf9 moth cells (*Spodoptera frugiperda*), which display spike protein on their cell membranes. Spike protein is incorporated into a nanoparticle of approximately 50 nm [76]. It is utilized for the immunization of individuals 18 years of age and older for preventing COVID-19. Novavax has signed a contract with the Serum Institute of India for large-scale manufacturing of the vaccine for providing to low-income developing countries by the brand name Covovax [77,78]. The dispersion injection of the vaccine consists of a 0.5 mL dose of 5 mg of the SARS-CoV-2 spike protein adjuvanted with *Quillaja saponaria* extract and Matrix-M, along with common excipients used widely. The vaccine may cause headaches, nausea, muscle aches, exhaustion, redness/soreness at the injection site, and fever [79].

Two-phase I/II trials were conducted on the vaccine, one in Australia (NCT04961541) and the other in the US (NCT04368988) with 642 and 1419 participants respectively. In Australia, the study aimed to assess the immunogenicity and safety of the vaccine along with a quadrivalent hemagglutinin nanoparticle vaccine. But, in the US the purpose of the study was to determine the efficacy of only the vaccine against COVID-19 by identifying the difference in effect with or without Matrix-M adjuvant. Then phase II trials were conducted in the Democratic Republic of the Congo, Kenya, Rwanda (PACTR202203582920839), South Africa (NCT04533399), and the United Kingdom of Great Britain and Northern Ireland (ISRCTN73765130). Furthermore, Serum Institute of India Private Limited has been conducting a phase II/III study in adults (18 years+) and children aged 2–17 years in India since 2 February 2021 (CTRI/2021/02/031554). Novavax is currently conducting a Phase III randomized, placebo-controlled study in collaboration with the Department of Health and Human Services to assess the safety, immunogenicity, and efficacy of the vaccine in 33,000 adult participants aged over 18 years, with a pediatric expansion of 12–17 years, since 27 December 2020, ending in 2023 (NCT04611802). Moreover, primary endpoint data from the study so far have revealed that the vaccine achieves infection prevention of about 90.4% in at least seven days after the second dose and 100% effectiveness against moderate-to-severe conditions [80]. NVX-CoV2373 was both safe and effective in preventing COVID-19. The majority of breakthrough cases were caused by modern variant strains (NCT04611802) [81]. According to Raburn M Mallory and colleagues [82], “This secondary analysis of phase 2, the randomized study assessed a single booster dose of a SARS-CoV-2 recombinant spike protein vaccine with Matrix-M adjuvant (NVX-CoV2373) in healthy adults aged 18–84 years, recruited from 17 clinical centers in the USA and Australia. Eligible participants had a BMI of 17–35 kg/m^2^ and, for women, were heterosexually inactive or using contraception. Administration of a booster dose of NVX-CoV2373 resulted in an incremental increase in reactogenicity. For both the prototype strain and all variants evaluated, immune responses following the booster were similar to or higher than those associated with high levels of efficacy in phase 3 studies of the vaccine. These data support the use of NVX-CoV2373 in booster programs”.

### 4.3. Biological-E Vaccine

CORBEVAX^TM^ SARS-CoV-2 (Biological-E) vaccine is a recombinant protein subunit vaccine. The vaccine is formulated from the virus’s spike protein harvest from cultured yeast *Pichia pastoris* and is used in combination with adjuvants like aluminum hydroxide gel and CpG 1018 [83]. It is only available in India for the emergency use category for COVID-19. Baylor College of Medicine and Texas Children’s Hospital Center in Texas, Houston, created the vaccine, which employs Dynavax technology. The license for its research and manufacture is now held by Biological E. Ltd. (BioE) [84]. The vaccine is intended for use in individuals above 12 years. The second dose of the vaccine is administered at least 4 weeks after the first dosage, and the related adverse effects in some individuals may include discomfort at the injection site, headache, high temperature, tiredness, nausea, and myalgia [85]. All the trials till phase III have been conducted in India, with 360 participants in phase I/II (CTRI/2020/11/029032), and 1268 participants in phase II/III (CTRI/2021/06/034014). Currently, a phase III trial is ongoing with 2140 participants for assessing the vaccine’s immunogenicity and safety in individuals negative by RT-PCR test (CTRI/2021/08/036074). The endpoints are indicating approximately 90% efficacy of the vaccine against SARS-CoV-2 infection [86].

### 4.4. HGCO-19 (Genova)

HGC0-19 is an mRNA-based COVID-19 vaccine evolved by HDT Bio Corp. and Genova Biopharmaceuticals with active funding from the National Institutes of Health (NIH) through the Indian Department of Biotechnology and Indo-US Vaccine Action Program (VAP) [87]. The novel vaccine candidate uses a lipid inorganic nanoparticle (LION™) for enhancing the potency [88]. The side effects related to the vaccination have not yet been reported [89]. Phase I studies for estimating vaccine dosage range, safety, and immunogenicity was conducted on 72 healthy adult subjects (CTRI/2021/04/032688). Upon obtaining satisfactory data, phase II/III studies for evaluating the immunogenicity, tolerability, and safety of the vaccine candidate in 4400 participants have recently been initiated in India (CTRI/2021/09/036379) [90].

### 4.5. Inactivated Rabies Vector Platform (Bharat Biotech)

To reduce the time for the scale-up of a vaccine, researchers at the Jefferson Vaccine Center at Thomas Jefferson University developed the idea of a new COVID-19 vaccine candidate, CORAVAX™ by incorporating previously known vaccine i.e., killed rabies vaccine as a carrier for delivery of SARS-CoV-2 spike proteins. The benefit of this approach is that the carrier vaccine to be incorporated has already proven data on its safety and efficacy, making the manufacturing task less tedious with low-cost production. Moreover, the rabies vaccine has the add-on ability to be administered safely to children, pregnant women, and diverse populations with almost life-long protection [91]. It was decided to develop the vaccine in collaboration with Bharat Biotech, India [92]. The vaccine is currently in the pre-clinical (animal testing) phase and soon will be entering human phase I trials. A list of India’s contributions to COVID-19 vaccines is summarized in Table 2.

## 5. Supply Chain and Global Distribution

The introduction of COVID-19 immunizations does not indicate that the global pandemic has ended; rather, countries must now acquire sufficient COVID-19 immunization doses and work to ensure their effective distribution. Immunization progress has been slow in all but a few high-income countries (HICs), while authorities learn how to immunize whole communities in the scenario of a pandemic [107]. The majority of low- and middle-income countries (LMICs) have depended on the COVID-19 Vaccination Global Access (COVAX) Facility to get vaccines. COVAX intends to send enough doses to these countries to vaccinate 20% of their populations. Despite their tremendous knowledge from the Expanded Program on Immunization, LMICs are projected to have more obstacles and issues in rolling out vaccines than HICs (EPI) [108,109]. In the absence of global efforts to ensure equitable vaccination distribution, the country of residence will almost probably become the single most important factor influencing vaccine distribution. A strictly pragmatic argument for conceptualizing the distribution of vaccinations against the SARS-CoV-2 virus on a worldwide scale is that bringing a vaccine to market will need governments from throughout the world to work in collaboration with charitable entities and consortiums [110,111]. In practice, one will require to combine not just national governments, but also scale-up global governing organizations to assure the essential institutions, organizations, and technologies to create and distribute a projected vaccine promptly and successfully to everybody who needs it. For the vaccine to be introduced, a competent supply chain management team is required. Countries should develop COVAX teams by utilizing committees and working groups, but keep in mind that the target groups will differ from those in traditional new vaccine introduction projects. As a result, the committees must be enlarged to include additional relevant stakeholders.

When a vaccine is approved by health organizations in various countries and regions, it goes through a development cycle and a fulfillment phase. The vaccine’s accessibility will be heavily reliant on the reduction of impediments in both the production and delivery phases of the supply chain. While developing new vaccines and verifying their efficacy in humans is the major focus, it is also critical to recognize and address VSC problems to increase vaccination efficacy [112,113]. To guarantee that COVID-19 immunizations result in broad immunization, governments must develop evidence-based policies. This study investigates and categorizes COVID-19 VSC concerns to help in the fight against the global pandemic. Consideration of supply chain difficulties before the distribution of a vaccine to the public can benefit the design of an effective vaccination program. As a result, finding substantial barriers to the COVID-19 VSC is critical for a long-term VSC that can help nations throughout the world to escape from the pandemic. Dimensions of obtaining broad worldwide COVID-19 immunity by vaccines include ‘development,’ ‘dissemination,’ and ‘deployment.’

The Government of India (GoI) has established large task forces such as NEGVAC (National Expert Group of Vaccine Administration for COVID-19), which are overseen by Municipalities (at the level of District Magistrate/Collector, Municipal Commissioner) and up to the State Principal Secretary (Health) and Chief Secretary. There has been much thinking and debate on the implementation, including sourcing and distribution, as well as the order of priority for who receives the vaccination first. This, like in other nations, begins with frontline healthcare personnel, then moves on to the elderly (divided into those over 60 and those between 50 and 60), and eventually to the rest of the population, along with the process of obtaining licensed vaccinations, as well as end-to-end storage and transportation logistics [114,115]. The Co-WIN (Covid Vaccine Intelligence Network) digital network is used to track the enrolled beneficiaries and, like performing free and fair elections, may be controlled by the Task Force established by the GoI. The total number of Covishield^TM^ doses manufactured now exceeds 1.3 billion.

High-income nations have acquired a large fraction of available vaccine doses, establishing purchase agreements for enough vaccines to vaccinate their whole populations many times; hence, the availability of the vaccine is an important parameter to be considered especially for developed countries [116]. While some pharmaceutical corporations have reduced vaccination pricing for impoverished nations or pledged to sell without profit for the length of the pandemic (Oxford University-AstraZeneca), others have not. Although numerous developing nations have the potential to produce vaccines, intellectual property (IP) rights and restricted knowledge transfer continue to be obstacles to building local manufacturing capacity [117]. Even if enough vaccine doses were available for the whole world’s population, rollout in underdeveloped nations poses several problems in terms of human capability and infrastructure. Only a few constraints must be overcome, such as weak ownership by governments and other stakeholders, which leads to inadequate strategy execution and poor accountability for meeting Global Vaccine Action Plan objectives. It has the potential to accelerate vaccine coverage in pandemic scenarios like COVID-19 [118]. The immunogenicity and effectiveness of vaccines are determined by how they are packed, stored, produced, and delivered. Vaccines must be stored in the right cold chain; the cold chain must be properly checked, and vaccines must be utilized only within crucial periods following removal from the cold chain or when a multi-dose vial is punctured [119]. According to Forman and Colleagues “Methodological issues and communication errors in clinical trials can lead to unrepresentative data and can even fuel vaccine hesitancy. For example, some of the participants in the AstraZeneca/Oxford vaccine’s UK-based clinical trial received roughly half of their intended first dose, and additionally, the timing between first and second doses varied between study participants, leading to criticism of the company’s initial 90% efficacy claim because these levels were seen in those who did not receive the standard regimen [120]”. In the context of a global public health emergency, such abbreviated regulatory routes and expedited implementations are still frequently recognized as experimental actions that are one-of-a-kind. To maintain public faith in vaccinations, however, comprehensive transparency in all aspects of vaccine development is required [121].

India has contributed not only to vaccine development, but also to treatment development. Various drugs and vaccines have been utilized across the world for suppressing the SARS-CoV-2 virus and one such drug utilized in India is, 2-deoxy-D glucose (2DG). It was licensed for emergency use as an additional treatment for moderate to severe COVID-19 patients by the Indian Council of Medical Research (ICMR) and the Drugs Controller General of India (DCGI) on May 1, 2021, during the second wave of the epidemic in India [110,111]. 2-DG is a glucose molecule with a 2-hydroxyl group that has been substituted by a hydrogen atom. This modification restricts the drug from entering glycolysis and contributing to ATP production. The drug is to be delivered orally in sachet/powder form by dissolving it in water for the patients. It assembles in virally infected cells and inhibits virus development by interfering with energy generation and viral synthesis. It has also proven diagnostic abilities for assessing the possibility of cancer by attaching a radioactive substrate [122,123,124,125].

Following the observation of satisfactory results in viral growth inhibition, INMAS-DRDO, in collaboration with Dr. Reddy’s Laboratories (DRL), Hyderabad, India, initiated a Phase II clinical trial, followed by a Phase III clinical study, with 110 and 220 patients enrolled respectively during May 2020 to assess the efficacy and safety of 2-DG as an adjunctive treatment against moderate to severe COVID-19 patients (CTRI/2021/01/030231). The results showed that hospitalized patients recovered faster with less need for supplementary oxygen once the medication was administered [123]. It still requires further justification for usage, which may be accomplished by undertaking multicentric research with a bigger and more diversified population [124,125].

## 6. Conclusions and Future Prospects

The COVID-19 global epidemic prompted an unbelievable joint project with significant financial investments from industry, academia, regulatory bodies, and governments [126]. India’s research groups likewise left no stone unturned in their quest for vaccine-preventative measures against COVID-19. Indian pharmaceutical businesses are one of the world’s leading vaccine makers, employing their capacity to the fullest, and are among world leaders in COVID-19 vaccine research. The entire globe is turning towards India, which, given enormous domestic needs and resource shortages because of a long lockdown (as for most other countries too), is not an only effort for greater forward progress but also going out of its way to fulfill its accountabilities to countries in need of help during these trying times. Furthermore, India is dedicated to educating medical practitioners from various economies on how to provide immunization safely and effectively. Vaccines thus far, have shown to be effective in preventing SARS-CoV-2 infection, and in the event of breakthrough infections, the risk of severe disease, hospitalizations, and death is considerably reduced [127]. In addition, due to the fast mutation rate of SARS-CoV-2, new vaccine efforts are required which are variant-specific. In addition, numerous antivirals have been approved and used in patients with severe COVID-19 disease. One such antiviral which has been developed in India is 2-deoxy-D-glucose (2DG).

A review of the emerging evidence about the necessity and timing of vaccination of children and adolescents with the COVID-19 vaccines has been conducted by WHO, in partnership with the Strategic Advisory Group of Experts on Immunization (SAGE) and the COVID-19 Vaccines Working Group. As part of their continuous review of the literature and continuing outreach to vaccine manufacturers, the research community, and the Member States, SAGE is seeking the most current, complete, and relevant information possible. An interim statement on maternal, newborn, child, adolescent, and perinatal health and nutrition was developed with additional support from the Strategic and Technical Advisory Group of Experts (STAGE) [128]. The potential risks and benefits of vaccination are not well known in pediatric populations.

To understand the benefits and risks of immunization, controlled clinical studies are required. Reduced severity, reduced mildness, or reduced transmission of disease might be the benefits. It has not yet been reported whether BBV152 has efficacy in children, but neutralizing antibodies were comparable to those measured in adult populations previously [129]. BNT162b2 (Pfizer-BioNTech) and mRNA-1273 (Moderna) are two mRNA-based COVID-19 vaccines approved for children by the EU and WHO. Regulatory authorities in the US and UK have also approved the use of BNT162b2 in children [130].

## Figures and Tables

**Table 1:** Various vaccines and the interval in-between vaccine doses.

Name of Vaccine *	Type of Vaccine	Indian Contribution	No. of Doses	The Interval between the First and Second Doses	The Time between the Second and Booster Dose	Reference
Covishield^TM^	Nonreplicating viral vector	Phase 3 trials and Manufacturing	2 doses	4–6 weeks (extended to 12–16 weeks)	24 weeks	[34,35]
Sputnik V	mRNA	Phase 3 trials and Manufacturing	2 doses	8 weeks	4 weeks	[36,37]
Covaxin	Inactivated	Fully developed and manufactured in India	2 doses	4–6 weeks	24 weeks	[38]
NVX-Co2373 (Novavax)	Protein subunit	Manufacturing	2 doses	3–4 weeks	2 months after 2nd dose; can be Pfizer- BioNTech or Moderna	[39]

* Sputnik V, Novavax, CovishieldTM, Covaxin, Corbevax, and ZyCoV-D are WHO-approved vaccines.

**Table 2:** India’s contribution against COVID-19 through vaccination.

Name	Route	Efficacy against Various Variants	Immune Responses	Clinical Trial Status	Ref.
BBV154 *	Intranasal	B.1.351 (Beta) B.1.1.28 (Gamma) B.1.617.1 (Delta)	Induces a vast immunological response, including IgG neutralization, T cell, and, mucosal IgA, responses.	Currently, Phase 3 trial is ongoing (NCT05522335)	[93,94,95]
CORBEVAX^TM^ SARS-CoV-2 (Biological-E) *	Intramuscular	B.1.351 (Beta), B.1.617.2 (Delta) B.1.1.529 (Omicron)	Cellular immune responses, anti-RBD-IgG1 titers, anti-RBD-IgG concentrations, and neutralizing Ab-titers.	Approved in 2 countries	[96,97,98]
COVAXIN *	Intramuscular	B.1.1.7 (Alpha) P.1-B.1.1.28 (Gamma) P.2-B.1.1.28 (Zeta) B.1.617 (Kappa) B.1.351 & B.1.617.2 (Beta & Delta)	It improves humoral and cellular immune responses	Approved, WHO Emergency Use Listing 14 Countries	[98]
Covishield^TM^	Intramuscular	B.1.1.7 (Alpha), B.1.617.2 (Delta)	SARS-CoV-2-specific IgG responses	Approved, WHO Emergency Use Listing 49 countries	[98,99,100]
Novavax	Intramuscular	B.1.1.7 (Alpha) B.1.351 (Beta)	Yields high antibody levels, resulting in substantial SARSCoV-2 protection	Approved, WHO Emergency Use Listing 6 countries	[98,101,102]
HGCO-19 (Genova) *	Nasal	B.1.1.7 (Alpha) B.1.351 (Beta) P.1 (Gamma) B.1.617.2 (Delta) B.1.1.529 (Omicron)	Potent T-cell responses	Approved in 1 country	[98,103,104]
ZyCoV-D *	Intradermal	B.1.617.2 (Delta)	Uses a fragment of the SARS-CoV-2 virus’s genetic code-DNA or RNA-to activate an immune response against its spike protein	Approved in 1 country	[98,105,106]

* Fully developed and manufactured in India.

## Data Availability

Not applicable.
